# *Bifidobacterium infantis* utilizes N-acetylglucosamine-containing human milk oligosaccharides as a nitrogen source

**DOI:** 10.1080/19490976.2023.2244721

**Published:** 2023-08-23

**Authors:** Shuqi Li, Xiaomeng You, Asha Rani, Ezgi Özcan, David A. Sela

**Affiliations:** a Department of Food Science, University of Massachusetts, Amherst, MA, USA; b Department of Microbiology, University of Massachusetts Amherst, Amherst, MA, USA; c Department of Nutrition, University of Massachusetts Amherst, Amherst, MA, USA; d Department of Microbiology & Physiological Systems and Center for Microbiome Research, University of Massachusetts Medical School, Worcester, MA, USA

**Keywords:** human milk oligosaccharides, microbiota, bifidobacteria, nitrogen metabolism, 2-oxoglutarate

## Abstract

*Bifidobacterium longum* subsp. *infantis (B. infantis)* utilizes oligosaccharides secreted in human milk as a carbohydrate source. These human milk oligosaccharides (HMOs) integrate the nitrogenous residue N-acetylglucosamine (NAG), although HMO nitrogen utilization has not been described to date. Herein, we characterize the *B. infantis* nitrogen utilization phenotype on two NAG-containing HMO species, LNT and LNnT. This was characterized through *in vitro* growth kinetics, incorporation of isotopically labeled NAG nitrogen into the proteome, as well as modulation of intracellular 2-oxoglutarate levels while utilizing HMO nitrogen. Further support is provided by comparative transcriptomics and proteomics that identified global regulatory networks deployed during HMO nitrogen utilization. The aggregate data demonstrate that *B. infantis* strains utilize HMO nitrogen with the potential to significantly impact fundamental and clinical studies, as well as enable applications.

## Introduction

Microbial colonization of the infant gastrointestinal tract plays a fundamental role in infant health, which is a critical window for gut maturation^1^ and immune system development^2^. Diet is widely recognized as an important determinant of gut microbiota colonization during infancy. Human milk is often the first food introduced to the infant and delivers oligosaccharides which transit intact to the colon as they are indigestible.^3^ These human milk oligosaccharides (HMOs) are utilized by the small fraction of gut commensals that are capable of doing so, to date mostly characterized as specific taxa within the genus *Bifidobacterium*.^4^ Accordingly, *Bifidobacterium longum* subsp. *infantis* (*B. infantis*) encodes a suite of HMO utilization genes typically arrayed in co-linear clusters, which is linked to its ability to utilize HMOs.^5–7^

Bacteria catabolize various nitrogenous molecules to ammonia which subsequently is synthesized into glutamate and glutamine, ultimately to incorporate nitrogen within the cell.^8^ A common bacterial system to accomplish this is the glutamine synthetase/glutamate synthetase (GS-GOGAT) pathway, which produces glutamate from ammonia and 2-oxoglutarate (2-OG).^8^ In some bacteria, however, an alternate ammonium assimilation pathway proceeds through glutamate dehydrogenase (GDH). The production of glutamate through GDH also requires ammonia and 2-OG as substrates.^8^ Ammonia is derived from nitrogenous molecules, and it is likely that *B. infantis* produces 2-OG from acetyl-CoA *via* citrate synthase, aconitase, and isocitrate dehydrogenase in its incomplete TCA cycle.^9^ 2-OG typically resides at the intersection of carbohydrate and nitrogen metabolism, as it provides the carbon skeleton for nitrogen assimilatory reactions in GS-GOGAT or GDH pathways.^10^

Intracellular 2-OG levels fluctuate according to nitrogen availability in *Escherichia coli* subjected to nitrogen starvation, with intracellular 2-OG levels increasing fivefold after ammonium is removed from the growth medium^11^ and decreases 20-fold when ammonium chloride is added.^12^ This inverse relationship is also exhibited by other microbes, including *Herbaspirillum seropedicae*, ^13^ *Cyanobacteria*, ^14^ and *Saccharomyces cerevisiae* ^15^. However, in *Bacillus subtilis*, there was no relationship between 2-OG and nitrogen accessibility.^16^ Due to the conservation of nitrogen assimilation pathways and the relationship between 2-OG and nitrogen in bacteria, we hypothesized that intracellular 2-OG levels in *B. infantis* are inversely related with how efficiently the cells utilize the nitrogen source. Therefore, 2-OG was investigated as a potential marker for the efficiency that *B. infantis* metabolizes nitrogen.

HMOs have been primarily studied as a prebiotic carbon source, although they incorporate nitrogen in the aminosugar residue *N*-acetyl glucosamine (NAG), and acidic HMO species incorporate *N*-acetylneuraminic acid (i.e., sialic acid).^3^ Whether these nitrogen-containing HMOs and/or their constiuents are utilized as a nitrogen source for *B. infantis* is currently unresolved. In this study, lacto-*N*-tetraose (LNT), the most abundant non-fucosylated neutral HMO species^17^, and its isomer, which vary by a single glycosidic bond, lacto-*N*-neotetraose (LNnT), was evaluated as a primary nitrogen source for *B. infantis* along with features of their metabolism at the system level.

## Results

### *B. infantis* utilizes HMO and constituent NAG as a nitrogen source

**Figure 1.**
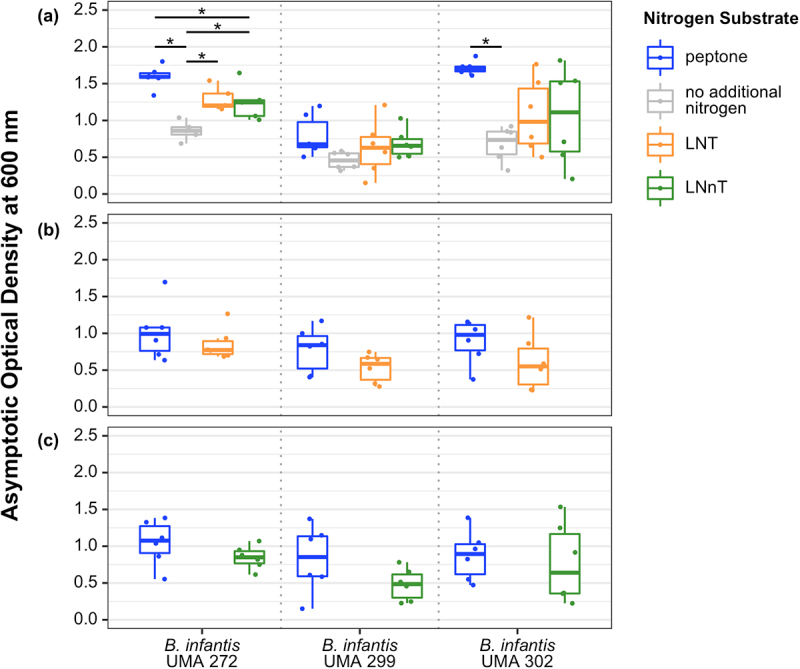
Terminal asymptotic optical density at 600_nm_ while *B. infantis* utilizes LNT or LNnT as a nitrogen source. Data were grouped into three panels according to carbohydrate conditions: (A) lactose, (B) LNT, and (C) LNnT. Adjusted *P* < 0.05 was designated significant.

*B. infantis* UMA 272 (parent strain ATCC 15697^T^) accumulated biomass while subsisting on LNT or LNnT as the primary nitrogen source which was significantly higher relative to the negative control (Figure 1A). Also, UMA 272 biomass while subsisting on LNnT was significantly lower compared to the positive control (i.e., peptone) (Figure 1A). Therefore, UMA 272 utilizes LNT or LNnT (albeit inefficiently) as a nitrogen source (Figure 1A). Despite differences in biomass accumulation, growth rates on LNT, LNnT, and peptone remained similar (Figure S1A). This suggests an equivalent preference for LNT, LNnT, and peptone as nitrogen sources.^18^ UMA 302 (parent strain JCM 7009) exhibited a higher growth rate while subsisting on either of the HMOs relative to the negative control (Figure S1A), although UMA 302 does not display appreciable growth beyond the negative control (Figure 1A). UMA 299 (parent strain JCM 1260), in contrast, does not accumulate any biomass or alter its growth rate while subsisting on HMO nitrogen. UMA299 is not an efficient HMO carbohydrate consumer and was analyzed specifically to characterize strain diversity. UMA299 often yields phenotypes inconsistent with other *B. infantis* strains, in particular, as it pertains to HMO utilization.

UMA 272 and UMA 302 achieved similar final biomass when utilizing LNT or LNnT as both the carbohydrate and nitrogen sources. This is in comparison to the positive control that had peptone as the nitrogen source and the corresponding HMO as the carbohydrate source (Figure 1B, 1C). This may indicate that LNT or LNnT could be utilized by *B. infantis* as a nitrogen source equivalent to peptone under specific conditions (e.g., while utilizing LNT as a carbon source).

Interestingly, when LNT is metabolized as the primary nitrogen source, trace amounts of NAG were detected in the supernatant for all strains (Figure S2A). This indicates that soluble NAG is secreted during LNT utilization in contrast to LNnT. Alternatively, it is possible that LNT is more available to extracellular hydrolysis, although this is contrary to what is currently understood regarding *B. infantis* HMO metabolism which proceeds intracellularly. Regardless, there is a clear phenotypic difference attributable to structural variation between the two tetrasaccharides. LNT and LNnT diverge solely by a terminal linkage between D-galactose and NAG at the non-reducing end, β-(1–3) and β-(1–4), respectively.

NAG is the nitrogen-containing residue in LNT and LNnT, thus *B. infantis* was evaluated for its ability to utilize the nitrogen from this soluble amino sugar. Accordingly, all three strains utilize NAG as a nitrogen source (Figure S3A). The growth rate is mixed among the strains with UMA 272 exhibiting a similar rate between NAG and peptone and UMA 302 displaying a reduced rate on NAG. Interestingly, UMA 299 had an elevated growth rate on 1% NAG and a depressed rate on 2%. In addition, *B. infantis* UMA 299 and UMA 302 exhibited higher consumption rates on 2% NAG nitrogen compared to UMA 272 (Figure S2B).

### ^15^N labeled NAG nitrogen is incorporated into the *B.* *infantis* proteome

**Figure 2.**
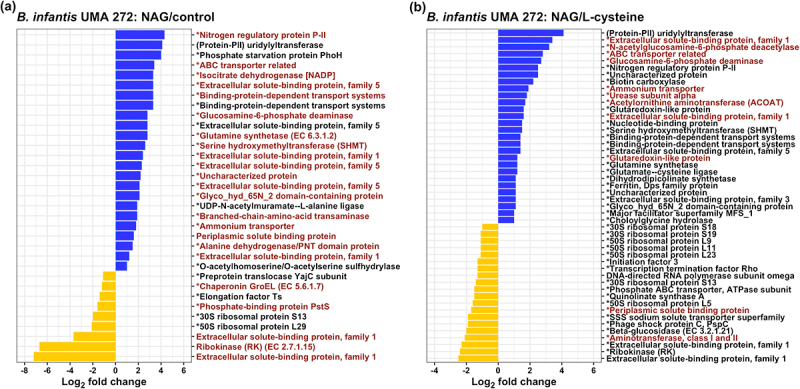
*B. infantis* differentially expressed proteins and genes while utilizing NAG as a nitrogen source. Differentially expressed genes during NAG utilization consistently within the transcriptome are colored in red. (A) differentially regulated proteins (DRPs) during NAG or complex nitrogen utilization. (B) differentially expressed proteins during NAG or L-cysteine utilization. Upregulated DRPs are colored in blue, and downregulated DRPs are colored in yellow. Asterisks indicate that the protein contain a ^15^N label. Log2 fold change (Log2fc > 1.5, *p* < 0.1 FDR corrected) values are indicated on x-axis.

In order to demonstrate the potential that HMO nitrogen incorporated in NAG is harnessed for anabolic reactions, ^15^N labeled NAG was tracked within the *B. infantis* proteome with isotopically labeled proteins analyzed by mass spectrometry. As anticipated, biological replicates clustered together (Figure S4A, S4B). ^15^N labeled NAG nitrogen was identified in Differentially Regulated Proteins (DRPs) in comparing NAG with either the complex control or L-cysteine (Figure 2, S5, S6). This approach efficiently quantified the fraction of the *B. infantis* proteome which only utilized ^15^N labeled NAG nitrogen beyond the contribution from L-cysteine to address cysteine auxotrophy.

^15^N derived from NAG was broadly distributed throughout the proteome, including core nitrogen metabolic processes. This includes glutamine/glutamate biosynthesis and urea catabolic proteins in addition to carbohydrate metabolism, cell wall biogenesis, ABC transporters, SBPs, and arginine biosynthesis. 433 proteins were identified while *B. infantis* utilized NAG or labeled NAG as a nitrogen source (Figure S5A). ^15^N was incorporated into 88% of the proteome (Figure S5A), other identified proteins (12%) included unlabeled NAG nitrogen only and thus were not included in the final incorporation analysis.

Furthermore,^15^N labeled L-cysteine was incorporated into the proteome in order to compare this with the ^15^N labeled NAG proteome (Figure S5B) as L-cysteine must be added to the growth media in limiting amounts. A minority of ^15^N labeled proteins 38.6% (n = 151) were shared among the NAG and L-cysteine proteomes with an additional 2.3% (n = 9) and 59.1% (n = 231) unique to ^15^N-labeled L-cysteine and ^15^N-labeled NAG proteomes, respectively (Figure S5B). Clearly, NAG nitrogen is catabolized as a nitrogen source and incorporated into the proteome. This is beyond the contribution that L-cysteine alone makes as a nitrogen source when it is added to address cysteine auxotrophy.

### Intracellular 2-OG concentrations increase while metabolizing LNT or LNnT as a nitrogen source

**Figure 3.**
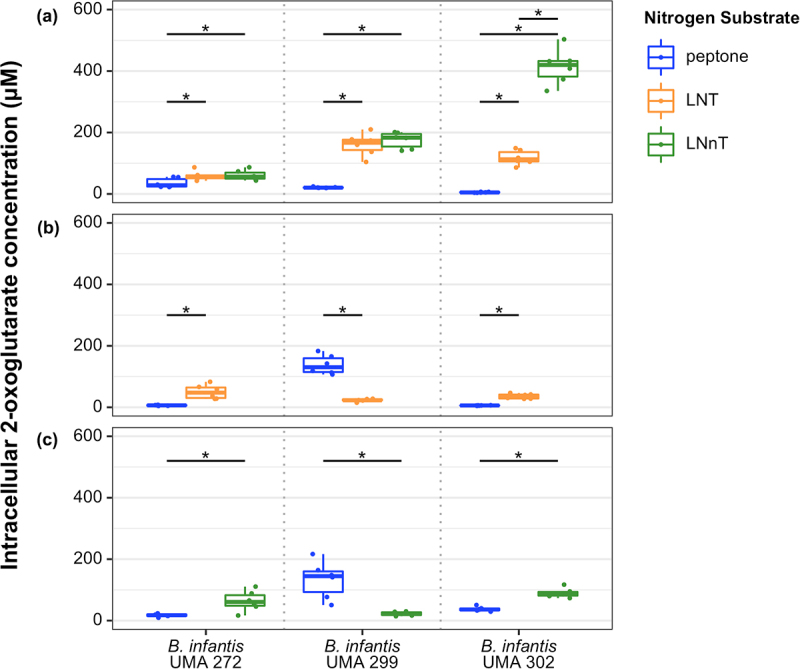
*B. infantis* intracellular 2-OG concentrations while *B. infantis* utilizes LNT, LNnT, or peptone nitrogen. Data are grouped into three panels according to carbohydrates fermented in the presence of a defined nitrogen: (A) lactose, (B) LNT, (C) LNnT. Adjusted *P* < 0.05 was designated significant.

Intracellular 2-OG concentrations were quantified while *B. infantis* strains utilized lactose as a carbohydrate source with LNT or LNnT as a nitrogen source. As a result, intracellular 2-OG concentrations were significantly higher during LNT or LNnT nitrogen utilization relative to the control in *B. infantis* UMA 272, UMA 299, and UMA 302 (Figure 3A). 2-OG concentrations were similar regardless of LNT or LNnT nitrogen utilization for strains UMA 272 and UMA 299. Interestingly, UMA 302 exhibited significantly higher concentrations of 2-OG while utilizing LNnT relative to LNT (Figure 3A).

Similarly, when LNT or LNnT served as the carbohydrate source and a nitrogen source (i.e., no lactose), UMA 272 and UMA 302 exhibited significantly higher 2-OG concentrations compared to peptone nitrogen (Figure 3B, 3C). In general, *B. infantis* intracellular 2-OG levels increased when LNT or LNnT was utilized as nitrogen source. The increased 2-OG concentrations are consistent with other bacterial systems that are enriched for this metabolism while utilizing an inefficient nitrogen substrate or while experiencing nitrogen starvation. Thus, there is evidence for an inverse relationship between 2-OG concentrations and nitrogen utilization efficiency in *B. infantis*.

### HMO carbohydrate metabolism does not impact intracellular 2-OG consistently

*B. infantis* utilizes LNT and LNnT as a carbohydrate source^19^, although it is unknown how this relates to intracellular 2-OG. Accordingly, it is known that 2-OG concentration is positively correlated with carbohydrate accessibility in *E. coli* ^10^, which contrasts with its inverse relationship with nitrogen availability. Interestingly, intracellular 2-OG levels were significantly lower while UMA 272 utilized LNT or LNnT compared to lactose (Figure S7A). In contrast, 2-OG levels significantly increased while *B. infantis* UMA 299 utilized LNT or LNnT (Figure S7A). In general, *B. infantis* UMA 299 is a poor HMO utilizer^6^, although it is unclear how this impacts flux through 2-OG pathways while utilizing HMO as a carbon or nitrogen source. UMA 302 increased 2-OG concentrations while utilizing LNnT as the carbohydrate source, although there was not any difference observed for LNT (Figure S7A). Interestingly, when LNT or LNnT was defined as the carbohydrate source and nitrogen source (i.e., no peptone and no lactose), 2-OG levels in UMA 299 and UMA 302 were significantly lower compared to cells grown on lactose (carbon) and HMO (nitrogen) (Figure S7B, Figure S7C).

### Secreted acetate to lactate ratio increases while metabolizing HMO nitrogen

*B. infantis* fermentation end-products during HMO nitrogen utilization were analyzed to characterize any deviation from typical metabolite profiles. HMO carbohydrate utilization has been previously studied, including secreted fermentation products.^19^ Contrasting with HMO carbohydrate metabolism, *B. infantis* only secreted acetate and lactate during HMO nitrogen utilization as formate and ethanol were not detected. *B. infantis* UMA 272 did not alter the acetate to lactate ratio relative to peptone nitrogen while metabolizing HMO nitrogen (Figure S2C). UMA 299 and UMA 302, however, increased the acetate to lactate ratio while utilizing LNT as a nitrogen source compared to peptone (Figure S2C). Interestingly, all strains exhibited a significant increase in the acetate to lactate ratio while NAG was utilized as a nitrogen source compared to peptone (Figure S2C). The increased ratio is likely due to additional acetate secretion through deacetylation of NAG and its residues within HMO.^20^

### The *B.* *infantis* transcriptome responds to HMO nitrogen utilization

The global transcriptomes of *B. infantis* strains were resolved while utilizing LNT, LNnT, NAG, and pooled HMO nitrogen. As expected, low sample-to-sample distances between two biological replicates for each nitrogen substrate were observed. Furthermore, the UMA 272 transcriptome while utilizing LNT nitrogen closely clustered with LNnT nitrogen metabolism (Figure S8A), which may be indicative of a similar *B. infantis* regulatory regime to harness nitrogen from these HMOs. *B. infantis* UMA 272 transcriptome profile during pooled HMO nitrogen utilizationclustered with complex nitrogen (Figure S8A), which indicates that pooled HMO serves as a sufficient complex nitrogen source for *B. infantis* UMA 272. Interestingly, the three *B. infantis* strains exhibited similar dendrogram topologies (Figure S8B, S8C, S8D). The transcriptome during NAG nitrogen utilization closely clustered with L-cysteine and divergent to complex nitrogen group regardless of strain (Figure S8B, S8C, S8D). This may be due to nitrogen metabolism regulation being more similar for the monomeric NAG and L-cysteine in contrast to oligosaccharides or the complex control.

### NAG catabolism is upregulated during HMO and NAG nitrogen utilization

Blon_0881 (*nagB*) and Blon_0882 (*nagA*) are essential for metabolizing NAG, and thus essential during HMO nitrogen utilization. Accordingly, Blon_0881 and Blon_0882 were significantly upregulated in the *B. infantis* UMA 272 transcriptome while LNT, LNnT, NAG, or pooled HMO was utilized as the primary nitrogen compared to the complex nitrogen control (Log2FC = 0.56–1.23, *p* < 0.001) (Figure S9, Table S1). This was verified by qRT-PCR targeting these two genes (Figure S10). In addition, Blon_0881 and Blon_0882 were significantly upregulated in UMA 272 when NAG was supplied as the primary nitrogen compared to L-cysteine (Log2FC = 1.13, *p* < 0.001) (Figure 2B, Figure S9, Table S1). *B. infantis* UMA 302 exhibited similar upregulation for Blon_0881 and Blon_0882 during NAG nitrogen utilization (Log2FC = 0.8–1.9, *p < 0.001*) (Figure S9, Table S1). The proteins encoded by Blon_0881 and Blon_0882 exhibited similar upregulation in the proteome (Figure 2, Table S2, S3). In summary, NAG catabolic genes and proteins are upregulated while *B. infantis* utilizes LNT, LNnT, NAG, and pooled HMO nitrogen.

### Nitrogen assimilation pathways are upregulated while utilizing NAG nitrogen

Gene and protein expression in pathways related to nitrogen metabolism were targeted, including the GS-GOGAT pathway, GDH pathway, and the PII nitrogen regulatory signaling (Figure 2, S9). In the GS-GOGAT pathway, glutamine synthetase (GS, Blon_1893 and Blon_0830) and glutamate synthetase (GOGAT, Blon_1481, and Blon_1482) facilitate the incorporation of nitrogen into glutamine and glutamate, respectively.^10^ Blon_1893 was significantly upregulated in UMA 302 (Log2FC = 5.04, *p < 0.001*) by NAG nitrogen utilization compared to the complex nitrogen control and to L-cysteine (Log2FC = 3.55, *p < 0.001*) (Figure S9, Table S1). Blon_1893 and Blon_0830 were significantly upregulated in *B. infantis* UMA 299 by NAG nitrogen compared to the complex nitrogen control (Log2FC = 2.90–3.82, *p < 0.001*). Blon_1481 and Blon_1482 were significantly upregulated in *B. infantis* UMA 302 under NAG nitrogen compared to the complex nitrogen control (Log2FC > 2, *p < 0.001*) and L-cysteine (Log2FC > 2, *p < 0.001*) (Figure S9, Table S1). The proteomics were consistent with this observation as glutamine synthetase (Blon_1893) was significantly upregulated by NAG compared to the complex nitrogen control in UMA 272 (Log2FC = 2.8, *p* < 0.05) (Figure 2A, Table S2) and compared to L-cysteine (Log2FC = 1.2, *p* < 0.05) (Figure 2B, Table S3). The other glutamate synthetase subunit (Blon_1481) was upregulated by NAG as compared to control but not significantly (log2FC = 2.5, *p* > 0.1) (data not shown). In general, the *B. infantis* GS-GOGAT system was upregulated while NAG was utilized as a nitrogen source.

In the GDH pathway, glutamate dehydrogenase (Blon_0011) incorporates ammonium into 2-OG to generate glutamate. Blon_0011 was significantly upregulated by NAG nitrogen compared to the complex nitrogen control in UMA 302 (Log2FC = 2.43, *p < 0.001*); however, it is significantly downregulated in UMA 299 (Log2FC = −2.25, *p < 0.001*) (Figure S9, Table S1) which may be a feature of this strain’s nitrogen utilization strategy favoring GS-GOGAT under these conditions. In addition, the protein glutamate dehydrogenase was not found to be significantly different under NAG nitrogen compared to control in proteomics profiles.

The PII nitrogen regulatory system involves an ammonium transporter (Blon_0223), PII protein (Blon_0224), and a uridylyltransferase (Blon_0225).^21^ Blon_0223 was significantly upregulated in *B. infantis* UMA 272 during NAG nitrogen and pooled HMO nitrogen utilization relative to complex nitrogen control (Log2FC > 2, *p < 0.01*) in UMA 299 and in UMA 302 compared to complex nitrogen control and L-cysteine (Log2FC > 2, *p < 0.001*) (Figure S9, Table S1). Blon_0224 was significantly upregulated in *B. infantis* UMA 272 and UMA 302 (Log2FC > 2, *p < 0.001*) during NAG nitrogen utilization compared to the control (Figure S9, Table S1). Blon_0225 was significantly upregulated in UMA 302 during NAG nitrogen utilization compared to the control (Log2FC > 2, *p < 0.001*) (Figure S9, Table S1). The upregulated PII nitrogen regulatory gene levels were consistent with the proteomics results. In the proteomics profiles, an ammonium transporter (Blon_0223), PII protein (Blon_0224), and uridylyltransferase (Blon_0225) were upregulated by NAG as compared to complex nitrogen control (log2FC > 1.8, *p* < 0.1) and L-cysteine (log2FC > 1.9, *p* < 0.1) (Figure 2, Table S2, S3). In general, the PII nitrogen regulatory system of *B. infantis* was upregulated by NAG nitrogen utiilzation, according to transcriptomics and proteomics results.

### The *B.* *infantis* urease gene cluster is significantly induced by NAG nitrogen and HMO nitrogen

*B. infantis* ATCC 17930 (UMA 299) utilizes urea as a nitrogen source, due to the expression of urease gene cluster.^22,23^ The urease gene cluster (Blon_0104–Blon_0115) is conserved in *B. infantis* UMA 272 as well as other strains.^5,6^ The transcriptome and proteome were interrogated to ascertain whether the urease gene cluster is co-regulated by another nitrogen source from human milk. Accordingly, Blon_0104–Blon_0115 were significantly upregulated in all strains while utilizing LNT, LNnT, and NAG nitrogen compared to complex nitrogen control (Log2FC > 2, *p* < 0.1) or L-cysteine (Log2FC > 2, *p* < 0.1) (Figure S9, Table S4). The urease subunit alpha (*ureC*, Blon_0111) was significantly enriched in NAG nitrogen group compared to L-cysteine (Log2FC = 1.8, *p <* 0.05) (Figure 2B, Table S3) in the proteome.

In addition, acetylornithine 5-aminotransferase (ACOAT) involved in arginine biosynthesis was also significantly upregulated by NAG utilization as compared to L-cysteine (Log2FC = 1.7, *p* < 0.05) (Figure 2B, Table S3). The upregulation of glutamine synthetase, glutamate synthetase, PII system, urease, and ACOAT indicates that *B. infantis* co-regulates overlapping nitrogen utilization systems during HMO and potentially other milk nitrogen substrate metabolism.

## Discussion

**Figure 4.**
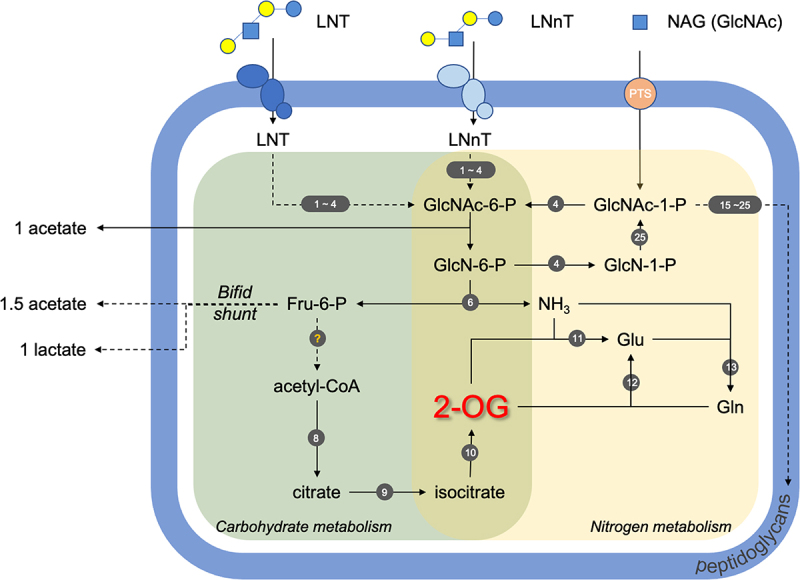
Model of *B. infantis* HMO nitrogen utilization. Nitrogen metabolism is shaded in yellow, carbohydrate metabolism in green, and peptidoglycan synthesis pathways in purple. Solid arrows are for one-step reactions and dashed arrows for multi-step reactions. The genes involved in the pathways are designated by numbers (1–25) in dark gray circles and listed in Table S5. LNT, lacto-*N*-tetraose; LNnT, lacto-*N*-neotetraose; NAG or GlcNAc, *N*-acetyl glucosamine; GlcNAc-6-P, *N*-acetyl glucosamine-6-phosphate; GlcNAc-1-P, *N*-acetyl glucosamine-1-phosphate; GlcN-6-P, glucosamine-6-phosphate; GlcN-1-P, glucosamine-1-phosphate; Fru-6-P, fructose-6-phosphate; 2-OG, 2-oxoglutarate; Glu, glutamate; Gln, glutamine; UDP-GlcNAc, uridine diphosphate-*N*-acetyl glucosamine; UDP-MurNAc, uridine diphosphate-*N*-acetyl muramic acid.

LNT and LNnT contain *N-*acetylglucosamine and thus we hypothesized that *B. infantis* harness these HMOs as a nitrogen source.^24^ Accordingly, *B. infantis* generates biomass on LNT or LNnT as a primary nitrogen source as well as incorporates NAG nitrogen into the proteome. The type strain *B. infantis* ATCC15697^T^ (UMA 272) exhibits the growth phenotype most prominently, and all strains exhibit hallmarks of utilizing HMO nitrogen including NAG nitrogen metabolism, transcriptome responses, and modulating intracellular 2-OG levels. UMA 299 was evaluated as a control as this strain does not efficiently utilize HMO, and the growth phenotype is a reflection of this, at least in part. UMA 302 was selected to investigate strain-variation among HMO utilizers, as it diverges in its utilization phenotype from the type strain. Growth phenotypes are a crude assay and the negative control (i.e., no additional nitrogen) is confounded by the L-cysteine auxotroph^25–27^ utilizing this essential amino acid as a nitrogen source as well, despite exhibiting metabolic hallmarks of HMO nitrogen utilization. The aggregate evidence demonstrates that *B. infantis* utilizes HMO as a nitrogen source with potential inter-strain variation. Figure 4 depicts a model of HMO nitrogen utilization that is supported by data presented herein.

We hypothesized that *B. infantis* intracellular 2-OG concentration is inversely related to the efficiency of nitrogen utilization as observed in other microbial systems. Nitrogen efficiency is defined herein as nitrogen that is less accessible (e.g., requires more cellular resources) that will yield inefficient growth compared to more preferred nitrogen substrates. Accordingly, *B. infantis* UMA 272 increased intracellular 2-OG concentration while inefficiently utilizing LNT or LNnT as a primary nitrogen source. *B. infantis* UMA 299 and UMA 302 also increased intracellular 2-OG concentration while utilizing LNT or LNnT nitrogen. The same phenotype was not observed while utilizing these HMO tetrasaccharides as a carbohydrate source, further supporting the conclusion that *B. infantis* utilizes HMO nitrogen. Interestingly, UMA 302 exhibited significantly higher 2-OG concentration while metabolizing LNnT nitrogen relative to LNT, which is indicative of strain-specific phenotypes that are related to the single glycosidic linkage difference between LNT and LNnT^19^. It is clear that *B. infantis* intracellular 2-OG concentration is inversely related to nitrogen utilization efficiency through comparison to the complex nitrogen control. These results are consistent with *E. coli* intracellular 2-OG that fluctuates with nitrogen source or availability as 2-OG levels are higher in nitrogen-limiting conditions.^10,28^

Utilization of NAG-containing HMO as a nitrogen source is supported by the incorporation of ^15^N labeled NAG into the *B. infantis* UMA 272 proteome, often into core nitrogen regulation processes. Accordingly, NAG deacetylase and deaminase were upregulated while utilizing LNT, LNnT, NAG, and pooled HMO as a nitrogen source as characterized in the transcriptome and proteome. Furthermore, isocitrate dehydrogenase, which facilitates the synthesis of 2-OG from isocitrate, was upregulated while utilizing HMO nitrogen. This is consistent with the molecular mechanisms involved in increasing intracellular 2-OG concentrations while utilizing HMO nitrogen. *B. infantis* nitrogen assimilation was upregulated in the transcriptome including glutamine synthetase and glutamate synthetase which incorporate nitrogen into the 2-OG carbon skeleton.

Interestingly, urease genes and proteins were upregulated under HMO nitrogen conditions. This suggests that the *B. infantis* urease gene cluster is co-regulated with ammonium assimilation and/or other nitrogen metabolism genes. Previous work reported that urease genes are co-regulated with glutamate/glutamine synthetase by a master regulator in *Streptomyces coelicolor* ^29^ (GlnR) and in *Corynebacterium glutamicum* ^30^ (AmtR) nitrogen metabolism. That these two divergent species also belong to the phylum *Actinobacteria*, suggest that *B. infantis* may possess a similar master regulator.

This work represents a significant advance in the definition of the *B. infantis* HMO utilization phenotype(s) and nitrogen metabolism in general. Specifically, it is now understood that *B. infantis* strains utilize HMO nitrogen that is shunted into anabolic processes *via* intracellular 2-OG among other features in the proteome and transcriptome. This supports further research to evaluate these processes *in vivo* within the infant gut microbiome. Moreover, this enables innovation of strategies to impact infant health and nutrition early in life, as it pertains to nitrogen cycling within the microbiome. This includes the rational design of synthetic milk formulas and other applications that specifically target bifidobacterial metabolism beyond carbohydrates.

## Material and Methods

### Routine propagation of bacterial cultures

*Bifidobacterium longum subsp. infantis* UMA 272 (ATCC 15697), UMA 299 (JCM 1260), UMA 302 (JCM 7009) were routinely propagated on de Man, Rogosa, and Sharpe (MRS; BD Difco) and supplemented with L-cysteine hydrochloride (0.05%, *w/v*, Across Organics) at 37°C under anaerobic conditions (Coy Laboratory Products). The anaerobic gas condition was maintained at a composition of >5% CO_2_, >5% H_2_, and the remainder as N_2_.

### Bifidobacterial *in* *vitro* growth

Single colonies were precultured in MRS broth (0.05% *w/v* L-cysteine) overnight. This was inoculated 1% (*v/v*) to experimental media which contained a defined carbohydrate source and a defined nitrogen source. Carbohydrate sources were limited to lactose (2%), LNT (2%), and LNnT (2%), while nitrogen sources were limited to NAG (1% or 2%), LNT (2%), LNnT (2%), pooled HMO (2%), or complex nitrogen control (2%). The experimental media shared a basic composition, including 0.2% (*w/v*) potassium phosphate, 0.02% (*w/v*) magnesium sulfate, 0.005% (*w/v*) manganese sulfate, 0.1% (*v/v*) Tween 80, and 0.03% (*w/v*) L-cysteine. 0.5% (w/v) sodium acetate was added when NAG was tested as the nitrogen source with lactose as the carbon source. The complex nitrogen control contained either 2% peptone or a combination of 1% peptone, 0.5% yeast extract, and 0.5% ammonium citrate. Each experimental group was evaluated in six biological replicates (at least) with technical triplicates. NAG was evaluated in biological triplicates. Growth kinetics were determined using the Growthcurver^31^ package in R^32^. The asymptotic optical density at 600_nm_ (asym OD_600nm_) and the growth rate constant of the first growth phase were extracted from the results for comparisons.

### Quantification of the intracellular intermediate metabolite 2-OG

*B. infantis* was inoculated from MRS preculture (0.03% L-cysteine) to the modified MRS (0.03% L-cysteine supplemented) media of defined carbohydrate and nitrogen conditions, each at 2% (*w/v*). Carbohydrate conditions were lactose, LNT, or LNnT and nitrogen conditions were peptone, LNT, or LNnT. The conditions are further defined in Table S6. The bacterial cells were harvested mid-log with OD_600nm_ measured by a Nanodrop 2000 (Thermo Fisher Scientific Inc.). Cells were harvested by centrifugation at 5000 rpm for 5 min. The resultant pellets were vortexed and incubated in 300 µL of 80:20 methanol:water over dry ice (−75°C) for 15 min (with 50 µL 0.1 mM ^13^C_5_-labeled 2-OG water solution spiked in). These samples were centrifuged (5000 rpm, 5 min, 4°C), and the supernatant was collected. The extraction was repeated twice. The final extract was concentrated, redissolved in a solvent system (1 mL) matching the mobile phase condition used in the liquid chromatography, filtered (0.22 µm, Sartorius), and stored at 4°C before injection into a UPLC/Xevo-TQD QQQ-MS system (Waters). The LC-MS method was adapted from Bajad et al.^33^ and validated for the instrument. Unlabeled 2-OG (Sigma-Aldrich) and ^13^C_5_-labeled 2-OG (Cambridge Isotope Laboratories, Inc.) were used as standards. Liquid chromatography was performed on an ACQUITY UPLC BEH Amide Column (130Å, 1.7 µm, 2.1 mm ×150 mm, Waters). Flow rate: 0.15 mL/min. Inject volume: 10 µL. Column temperature: room temperature (25 ± 2°C). The LC solvents used were Phase A: 20 mM ammonium acetate +20 mM ammonium hydroxide in 95:5 water: acetonitrile, pH 9.45; Phase B: acetonitrile. The gradients were as follows: *t* = 0 min, 85% B; *t* = 5 min, 0% B; *t* = 10 min, 0% B; *t* = 10.01 min, 85% B; *t* = 20 min, 85% B. Areas under peaks were used to interpret 2-OG concentration in samples. The retention time of both molecules was at 6.6 ± 0.57 min of each run. Finally, the measured 2-OG concentration was normalized by the corresponding OD _600 nm_ at the time of harvest to yield the final 2-OG concentration.

### Characterization of bifidobacterial extracellular metabolites

The concentration of lactate, acetate, formate, ethanol, and NAG in the spent media were determined by high-performance liquid chromatography (HPLC) analysis. Samples were prepared from the supernatants of the 96-well microplate, at early stationary phase under defined nitrogen conditions in mMRS media. Nitrogen sources were either 2% LNT, 2% LNnT, 2% NAG, or 2% peptone. Lactose was used as the carbohydrate source 2% (*w/v*) for all experimental groups. The cell-free supernatants were filtered through a 0.22 µm filter (Sartorius) and stored at −20°C until analysis. Substrate residues and organic acids were quantified using a Shimadzu HPLC system equipped with a Refractive Index Detector 20A (Shimadzu Corp.). The separation was carried out on an Aminex HPX-87 H column (7.8 mm ID × 300 mm, Bio Rad Laboratories) at 30°C. The mobile phase was 5 mM H_2_SO_4_, flow rate was 0.6 ml/min, and the injection volume was 25 μL. Lactate, acetate, and NAG concentrations were calculated from standard curves (0.5, 1, 5, 10, 20, and 50 mM). Four biological replicates were performed for each experimental condition.

### Quantitative real-time PCR to measure relative gene expression

One ml samples were harvested at mid-exponential phase (OD_600nm_ ~0.4–0.6), pelleted at 12,000 × g for 2 min, and stored in 1 ml Ambion RNAlater (Life Technologies). RNA extraction and cDNA conversion were performed as previously described.^34^ Samples were centrifuged at 12,000 × g for 2 min to collect the cell pellet. The pellet was washed twice with PBS buffer to remove residual RNAlater and centrifuged at 12,000 × g for 2 min. Total RNA was extracted using Ambion RNAqeous-Mini kit (LifeTechnologies) according to the manufacturer’s instructions. Cells suspended in lysis buffer were transferred to Lysing Matrix E tubes (MP Biomedicals LLC) to disrupt cell walls through bead beating at 5.5 m/s for 30 s twice using FastPrep 24 bead beader (MP Biomedicals). Total RNA was eluted in 50 μl of EB solution and immediately subjected to DNase treatment with the Ambion Turbo DNA-free (Invitrogen) using 1 μL of DNase I for 30 min. Subsequently, total RNA was converted to cDNA using the High-Capacity cDNA Reverse Transcription Kit (Applied Biosystems) according to the manufacturer’s instructions. The resultant cDNA was quantified by a Nanodrop 2000 Spectrophotometer (Thermo Fisher Scientific Inc.). The qRT-PCR was performed on a 7500 Fast Real-Time PCR System (Applied Biosystems) with PowerUP SYBR Green Master Mix (Applied Biosystems, Foster City, CA) using 200 ng of input cDNA. The reaction conditions were optimized for the specific target locus. qRT-PCR primers were designed using the Primer3 software (Table S7; http://frodo.wi.mit.edu). The gene Blon_0393, encoding a cysteinyl-tRNA synthetase was used as an endogenous control as previously.^34,35^ Growth on lactose (2% wt/v) served as a reference condition for gene expression. Results were expressed as fold change relative to the reference. These experiments were conducted on biological triplicates, and triplicate technical measurements were performed. Following DNase treatment, the absence of genomic DNA was confirmed using the total RNA as template by qRT-PCR targeting the endogenous control.

### Transcriptome analysis in response to human milk nitrogen sources

#### RNA isolation and purification

Bacteria were grown in mMRS media supplemented with pooled HMOs (2%, *w/v*), LNT (2%), LNnT (2%), NAG (2%), complex nitrogen (1% peptone, 0.5% yeast extract, 0.5% ammonium citrate), or L-cysteine (0.15%) as the nitrogen source, while 2% lactose as supplemented as the carbohydrate source. Bacteria cells were harvested at exponential phase and pelleted immediately by centrifugation at 12,000 × g for 1 min and resuspended in 1 ml of RNAlater Ambion (Thermo Fisher Scientific) and incubated at 4°C overnight and then stored at −80°C until further analysis. RNA isolation was performed using an Ambion RNAqueous^TM^ kit (Thermo Fisher Scientific) following manufacturer’s instructions. An additional step of bead-beating for cell disruption (FastPrep-24TM 5 G, MP Biomedicals Inc) was added to the RNA isolation procedure. Total RNA was immediately treated with the Turbo DNase free kit (Ambion, Thermo Fisher Scientific). Samples with an RNA integrity number (RIN) above 7 were selected for further processing, as checked by the High Sensitivity RNA analysis ScreenTape Assay (Agilent Technologies). DNA contamination was assessed by qRT-PCR using the Fast SYBR Green master mix (Applied Biosystems) with the primers: Uni334F, 5’-ACTCCTACGGGAGGCAGCAGT-3’ and Uni514R, 5’-ATTACCGCGGCTGCTGGC-3’).

#### Library construction and sequencing

Total RNA was treated with Ribo-Zero rRNA Removal kit for Bacteria (Illumina) to remove ribosomal RNA according to manufacturer’s instructions. The depleted RNA was purified with the RNAeasy MinElute Cleanup kit (Qiagen). The rRNA depletion efficiency was evaluated with the High Sensitivity RNA analysis ScreenTape Assay (Agilent Technologies). Whole transcriptome libraries were constructed using TruSeq Stranded mRNA Library Preparation Kit (Illumina) or NEBNext Ultra II Directional RNA library Prep Kit for Illumina (New England Biolabs Inc) following manufacturer’s instructions. Barcoded library quality was assessed by DNA analysis ScreenTape Assay (Agilent Technologies) and quantified by Qubit dsDNA BR Assay (Life technology). Libraries were diluted and pooled to equimolar concentration and denatured immediately prior to sequencing on an Illumina NextSeq 500 sequencing system (150 cycles, paired-end sequencing, two runs). Raw reads and processed data were publicly deposited in the NCBI Gene Expression Omnibus database (https://www.ncbi.nlm.nih.gov/geo/) under accession number GSE209665 and GSE155078.

#### Quality control, alignment, and gene counting

Raw reads were uploaded to the Massachusetts Green High-Performance Computing Cluster used for all bioinformatic and statistical analyses unless specifically noted. The raw read quality was checked by FastQC^36^ (v1.0.0). For libraries prepared by NEBNext Ultra II Directional RNA library Prep Kit, the sequencing adapters were removed by Trimmomatic^37^ (v0.32). For libraries prepared by TruSeq Stranded mRNA Library Preparation Kit, the sequencing adapters were trimmed with the Illumina FASTQ generation pipeline. The quality-controlled and trimmed reads were then aligned to the *Bifidobacterium longum* subsp. *infantis* ATCC 15697^T^ genome (NCBI accession number: NC_011593.1) using bowtie2^38^ (v2.1.0). The aligned reads were sorted by SAMtools^39^ (v1.0) and counted against a specific genomic locus using HTSeq^40^ (v0.6.1) for differential expression analysis.

#### Differential gene expression analysis

Differentially expressed gene analysis was performed using the R Package DESeq2^35^ (v3.3.1). The gene count tables generated from HTSeq were used as inputs, and the inbuilt function was applied to identify and quantify the magnitude of differentially expressed genes. Whole transcriptome comparisons were analyzed by hierarchical clustering analysis. The results were visualized by DESeq2 and pheatmap^41^ (R package, v1.0.12). Log2 fold-change (Log2FC) of gene expression was calculated by inbuilt function in R Package DESeq2 and visualized using a pheatmap. Statistically significant differences were considered with Benjamini–Hochberg adjusted p-value (FDR) < 0.05.

### Proteomic analysis of bifidobacterial utilization of nitrogen

#### Proteome sample preparation

Bacterial cultures were grown in mMRS supplemented with NAG or complex nitrogen source or L-cysteine (0.03% w/v). *B. infantis* UMA 272 overnight culture was inoculated (1%) into 2.5 ml of mMRS (0.03% L-cysteine) with a defined nitrogen source. A total of three biological replicates were included for proteomic analysis from each ^15^N labeled and unlabeled nitrogen samples. Condition 1: Complex nitrogen control (2%), condition 2: ^N15^L-cysteine (0.03% w/v, HSCH_2_CH(*NH_2_)COOH, Cambridge isotope laboratories, Inc.), or unlabeled L-cysteine (0.03% *w/v*), condition 3: unlabeled L-cysteine (0.03%) + unlabeled NAG (2%), or ^N15^L-cysteine (0.03%) + unlabeled NAG (2%) or unlabeled L-cysteine (0.03%) + ^N15^NAG (2%, C8H15*NO6, Cambridge isotope laboratories, Inc.).

Cultures were harvested at mid-exponential phase and centrifuged at 12,000 × g for 3 min, washed 2× with PBS buffer and stored at −80°C until further analysis. The cell pellets from storage were suspended in 1X SDS lysis and solubilization buffer (Protifi LLC) then sonicated using Bioruptor pico (Diagenode) at high for 10–15 min (sonication cycle: 30 sec ON, 30 sec OFF). The cell lysate was clarified by centrifugation at 13,000 g for 8 min. Protein concentrations were measured using the Pierce BCA protein Assay kit (Thermo Fisher Scientific) on a Nanodrop spectrophotometer (Thermo Fisher Scientific). Samples were reduced, alkylated, and trypsin digestion, and clean-up was performed according to S-Trap micro universal MS sample prep kit (Protifi LLC). Briefly, each sample was mixed with digestion buffer containing mass spectrometry grade trypsin (Promega) at a 1:50 ratio (wt/wt) and was added to the micro-spin column. Tryptic peptides were then reconstituted and purified with ZIPTIP® C18 pipette tips (Merck Millipore).^42^

#### Mass spectrometry proteomics and data analysis

Tryptic digests were analyzed using a Thermo Easy-nLC 1000 nanoLC system coupled to a Thermo Orbitrap Fusion mass spectrometer (Thermo Fisher Scientific) at the University of Massachusetts Amherst Mass Spectrometry Center.

Mass spectrometry data was analyzed using Peaks studio software (V.10.5, Bioinformatics solutions Inc.). To identify peptides and infer proteins, a search was performed against a series of protein databases using the multi-step database strategy as implemented in Peaks Studio. In step 1, the UniProt protein database (www.uniprot.org) was searched using a *Homo sapiens* and *Mus musculus* filter along with a common contaminants database. Unmatched *de novo* tags from step 1 were queried against a database constructed from the UniProt database containing *Bifidobacterium longum* subsp. *infantis* ATCC 15697 (https://www.uniprot.org/proteomes/UP000001360, Proteome ID: UP000001360).^5^ A mass tolerance of 15 ppm was allowed for precursor ion and 0.5 Da for-fragment ions, with the peptide hitting a threshold of ≥25 (−10logP). The Peaks software package was used for qualitative analysis with the following parameters added: a peptide identification filter of 1% FDR for peptide-to-spectrum matches (PSMs) was set, and protein identification was based on at least one unique peptide per protein. For the database search, the following parameters were applied: (i) trypsin as enzyme for digestion, (ii) up to three missed cleavages per peptide, (iii) carbamidomethyl cysteine as fixed, (iv) methionine oxidation/acetylation (N-term), and ^15^N labeling on nitrogen as variable modifications. The FDR for peptide and protein identification was assessed using the target-decoy strategy by searching against the reverse database. Proteins and peptides were identified and quantified by at least two of the three replicates.

Multivariate analysis was carried out using MetaboAnalyst 4.0 (www.metaboanalyst.ca).^43^ In order to generate relative quantifications across samples abundances were normalized to the central mean based on the total sum of peptide and protein abundances and were log transformed prior to the fold change (Log_2_FC) analysis. Differentially expressed proteins (DEPs) were defined as proteins with a fold change >2.0 and with significance *p* > 0.1 (FDR corrected) and were visualized using volcano plots. Proteins were annotated with GO terms and KEGG pathways to identify biological functions using UniProt ID mapping.

### Statistical analysis for growth kinetics, qRT-PCR, and metabolites quantifications

Bacterial growth biomass, growth rate, and relative gene expression from qRT-PCR were subjected to one-way ANOVA followed by Tukey’s HSD Test. Acetate to lactate ratio data was subjected to a two-way ANOVA followed by Tukey’s HSD Test. Statistical tests were performed using base R or GraphPad Prism (version 9.0.0 for Mac OS X, GraphPad Software). Plots were generated using GraphPad Prism and R package tidyverse^44^.

## Supplementary Material

Supplemental Material

Supplemental Material

## Data Availability

Transcriptome reads and processed data are deposited in the NCBI Gene Expression Omnibus database (https://www.ncbi.nlm.nih.gov/geo/) under accession numbers GSE209665 and GSE155078. Proteome data is deposited in the EMBL-EBI PRoteomics IDEntifcations Database (PRIDE) under Accession: PXD035809
