# Supplementary material for: Bifidobacterium infantis utilizes N-acetylglucosamine-containing human milk oligosaccharides as a nitrogen source
Source: Gut Microbes. 2023 Aug 23;15(2):2244721. doi: 10.1080/19490976.2023.2244721 (PMC10448974; doi:10.1080/19490976.2023.2244721)
Supplement: Supplemental Material [file KGMI_A_2244721_SM2262.docx]

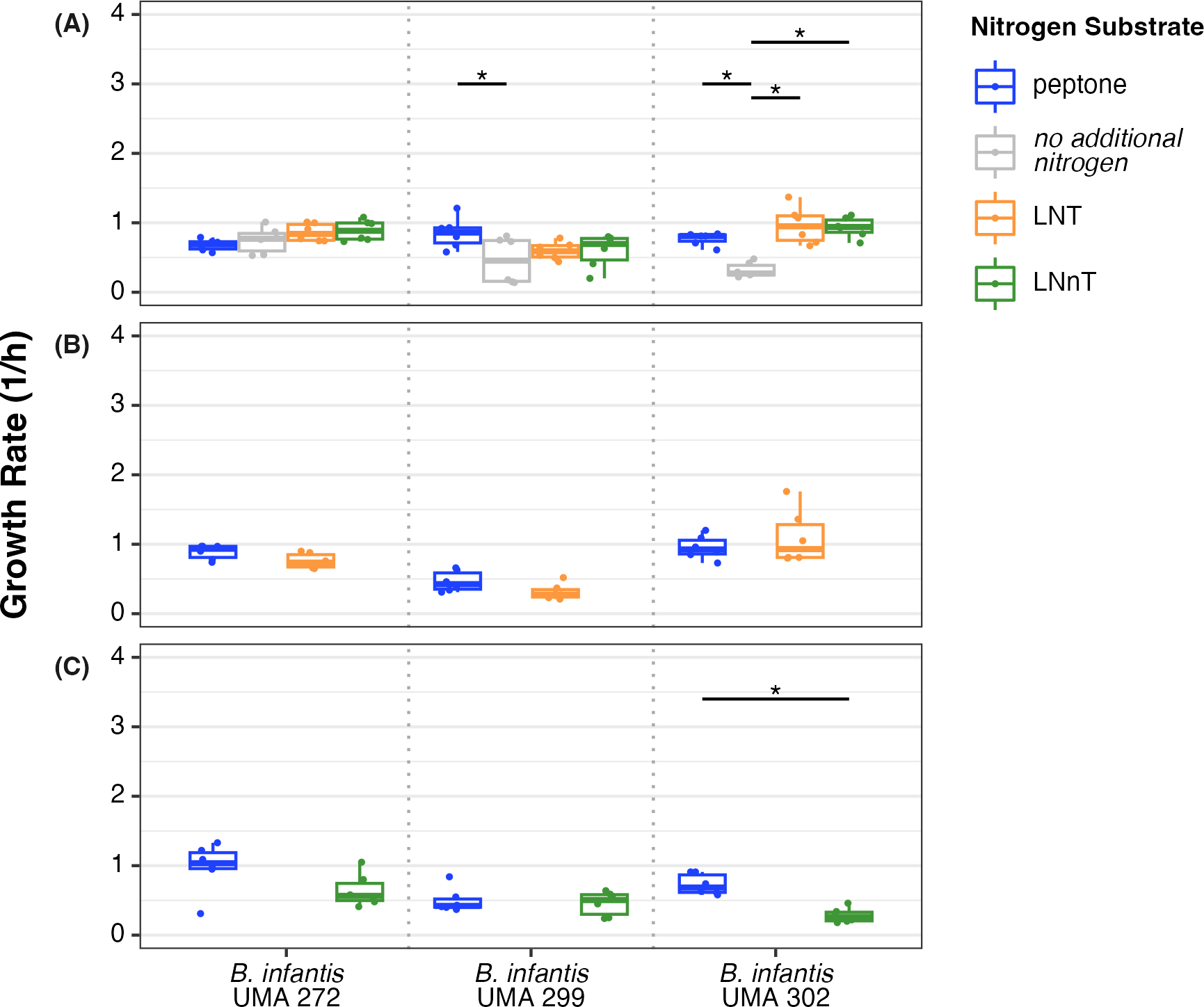


# Figure S1

*B. infantis* growth rates in LNT, LNnT, peptone, and no additional nitrogen conditions. Data were grouped into three panels according to *B. infantis* carbohydrate conditions: (A) lactose, (B) LNT, (C) LNnT. One-way ANOVA was performed among nitrogen conditions within same strain and same carbohydrate conditions. Tukey’s post- hoc test was performed for multiple comparisons between two nitrogen conditions. Adjusted P <0.05 * was regarded significant difference.

# Figure S2


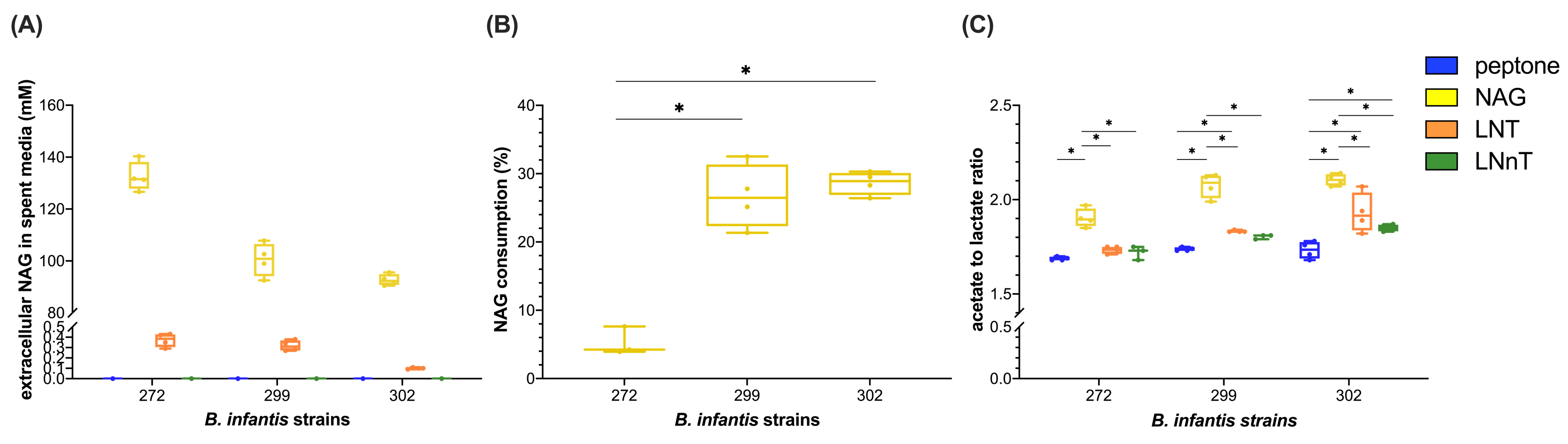
*B. infantis* extracellular metabolites in peptone, NAG, LNT and LNnT nitrogen conditions. (A) NAG consumption rate of

*B. infantis* UMA 272, UMA 299, and UMA 302 while NAG as supplied as the nitrogen source. (B) NAG

concentration in the spent media of *B. infantis* UMA 272, UMA 299, and UMA 302 (A) The acetate to lactate ratio of *B. infantis* UMA 272, UMA 299, and UMA

302. The carbohydrate condition of all the

groups was lactose. One-way ANOVA was performed among nitrogen conditions within same strain. Tukey’s post-hoc test was performed for multiple comparisons between two nitrogen conditions. Adjusted P <0.05 * was regarded significant difference.

#
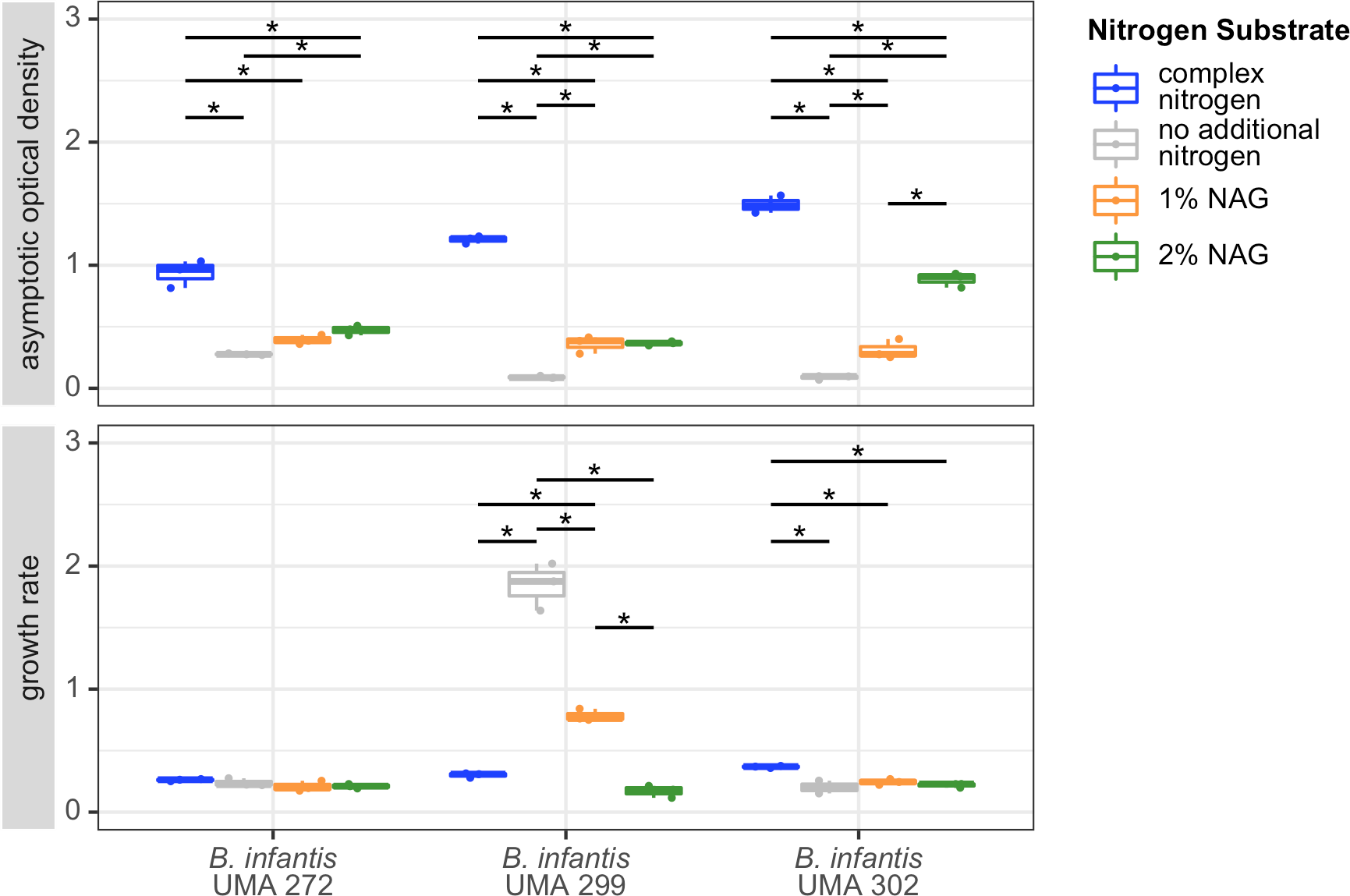
Figure S3

*B. infantis* asymptotic optical density at 600nm and growth rates in complex nitrogen, no additional nitrogen, 1% (w/v) NAG, and 2% (w/v) NAG nitrogen conditions. One-way ANOVA was performed among nitrogen conditions within the same strain and under same carbohydrate conditions. Tukey’s post- hoc test was performed for multiple comparisons between two nitrogen conditions. Adjusted P <0.05 * was regarded as a significant difference.

#
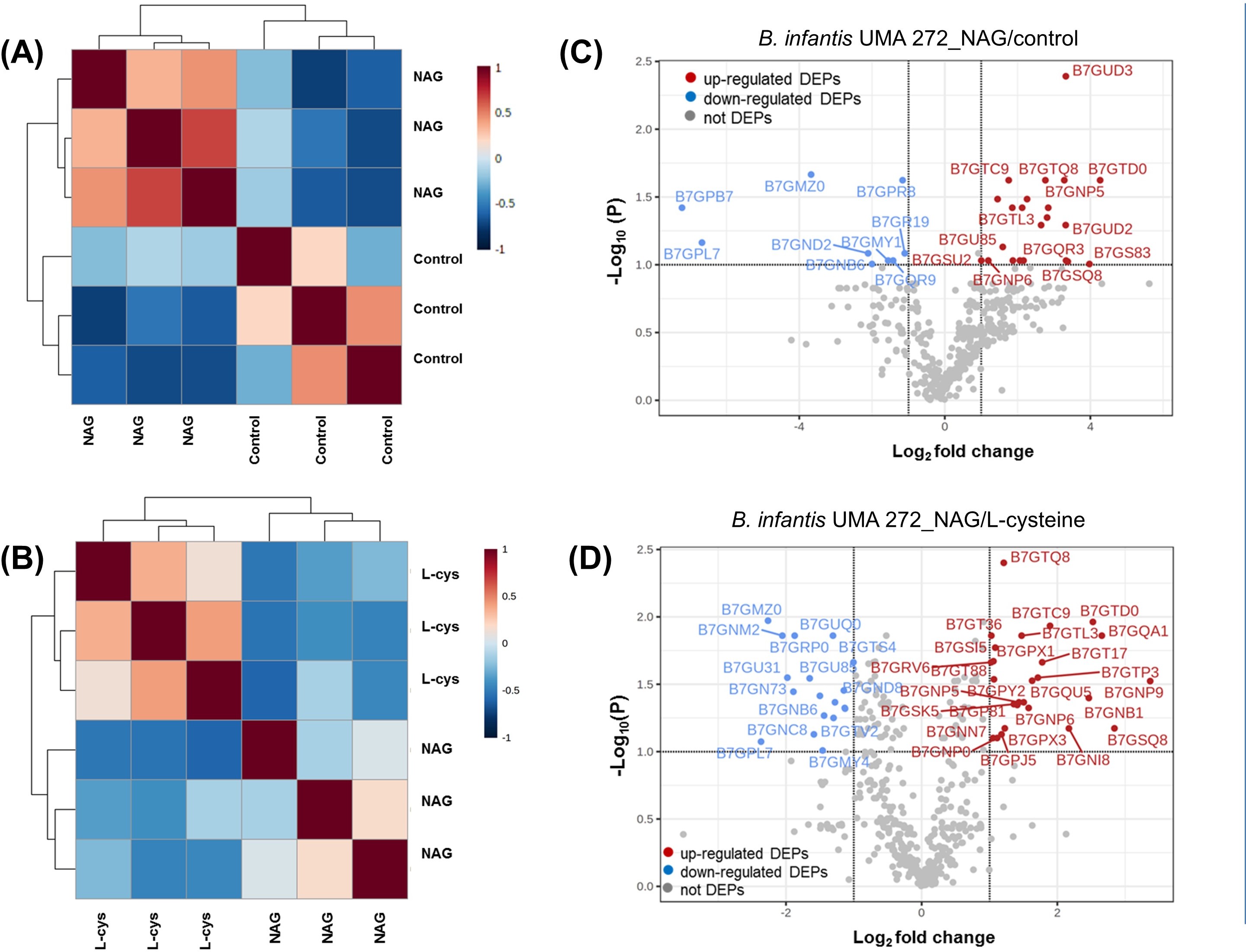
Figure S4

*B. infantis* UMA 272 protein expression is similar during growth on NAG as compared to (A) complex nitrogen (positive control) and (B) L-cysteine (negative control) as seen from correlation heatmaps (representing sample to sample distance). The color scale bar next to correlation heatmaps represents the Pearson correlation sample to sample distance score. Volcano plots showing differential expression of proteins (DEPs) during utilization of NAG as compared to (C) complex nitrogen (positive control) and

(D) L-cysteine (negative control). Proteins upregulated and downregulated are shown in red and blue respectively. Log2 fold change (FC >2.0) and −log 10 (p-value, FDR corrected) are shown on x and y-axis respectively.

# Figure S5


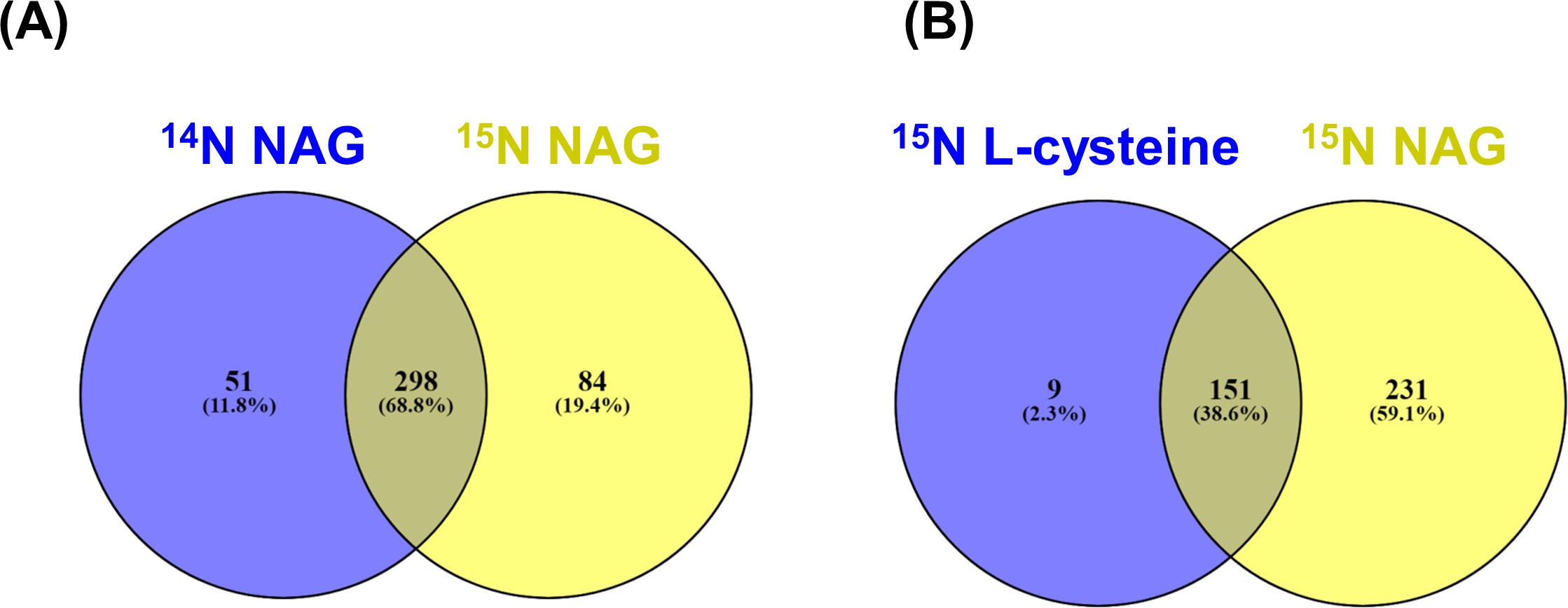
Venn diagram representations of 15N labeled proteins identified in *B. infantis* UMA 272 during NAG vs L-cysteine utilization. (A) Venn diagram representation of number of labelled proteins identified in *B. infantis* UMA 272 during utilization of NAG as primary nitrogen source. Approximately 69% proteins were shared among both datasets with additional 11.8% and 19.4% unique in 14N (blue) and 15N labelled (yellow) NAG respectively. (B) Venn diagram representation of number of 15N labeled proteins identified in B. infantis UMA 272 during utilization of NAG as primary nitrogen source compared to 15N labelled L-cysteine. Approximately 39% proteins were shared among both datasets with additional 2.3% and 59.1% unique in 15N labelled L-cysteine (blue) and 15N labelled NAG (yellow) respectively.

# Figure S6

General *B. infantis* proteome results without isotopic labels. (A) Venn diagram for number of proteins identified in *B. infantis* UMA 272 during utilization of NAG or complex nitrogen (control) as primary nitrogen source. Approximately 54% proteins were shared among both the datasets with additional 22% and 24% unique proteins to NAG and control group respectively. (B) Venn diagram for number of proteins identified in *B. infantis* UMA 272 during utilization of NAG or L- cysteine (negative control) as primary nitrogen source. Approximately 60% proteins were shared among both the datasets with additional 18% and 22% unique proteins to the NAG and L- cysteine groups respectively.

1.
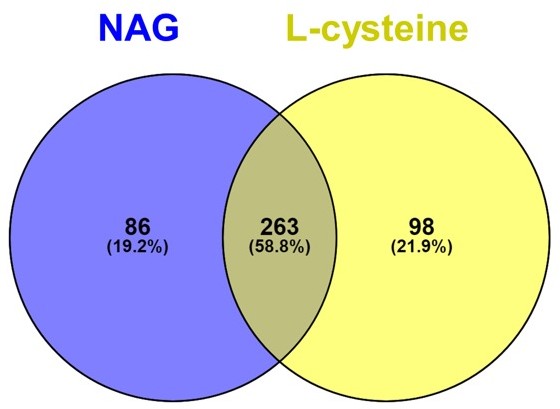

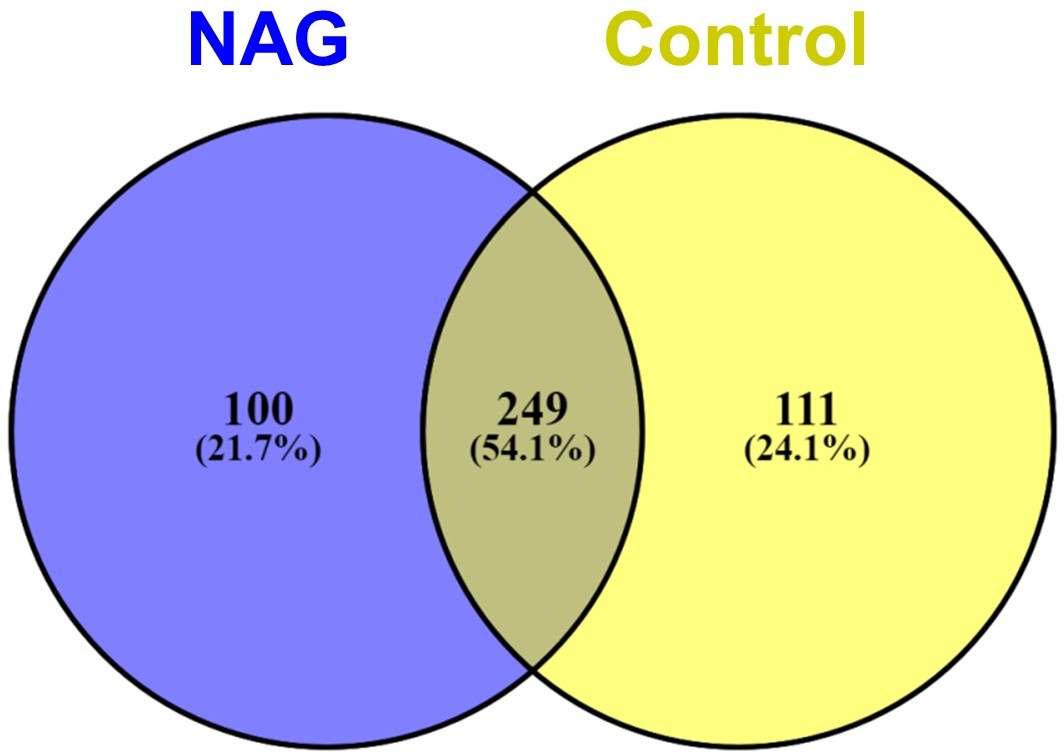
**(B)**

#
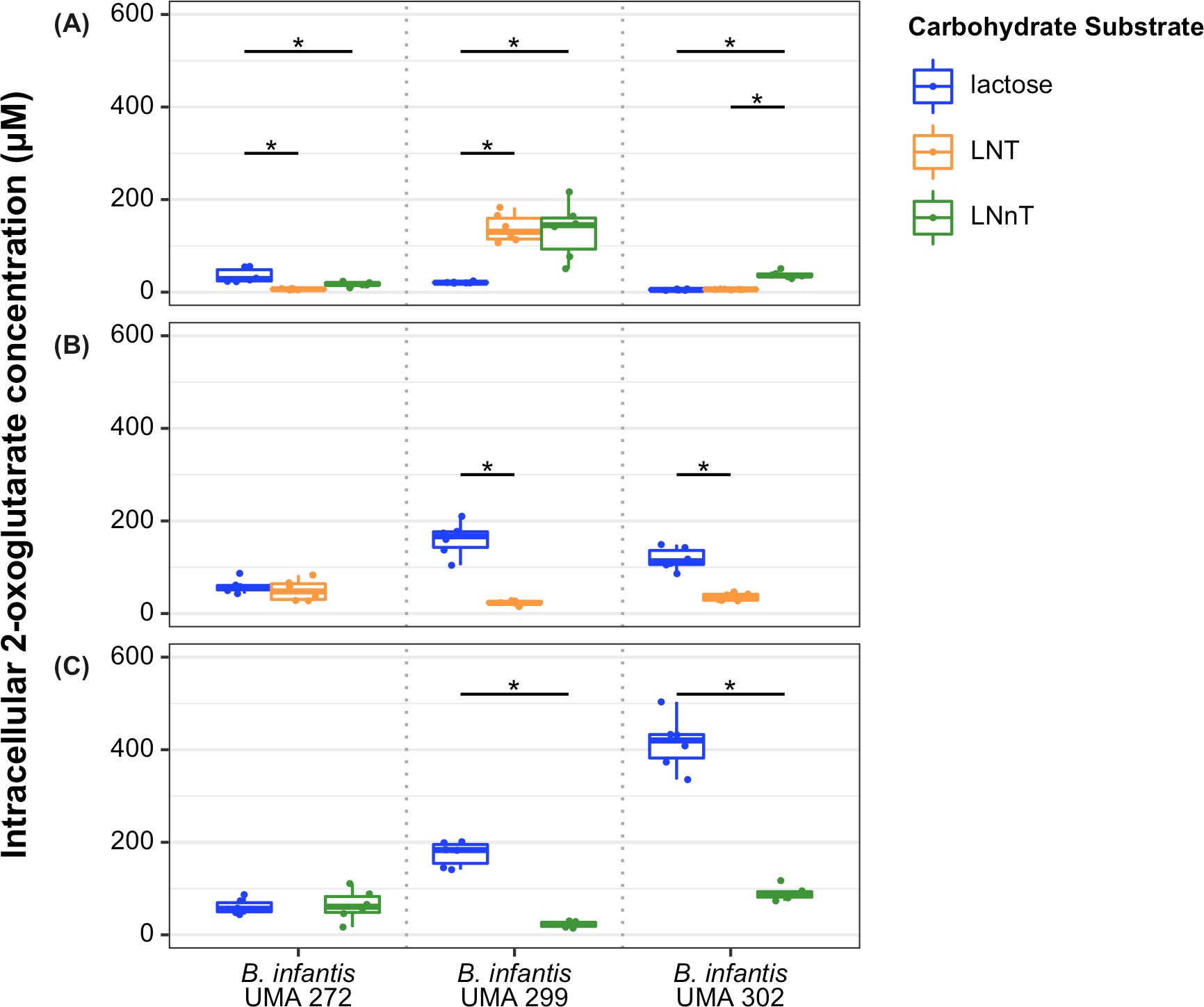
Figure S7

*B. infantis* intracellular 2-oxoglutarate (2- OG) levels in lactose, LNT, and LNnT carbohydrate conditions. Data were grouped into three panels according to *B. infantis* nitrogen conditions: (A) peptone,

1. LNT, and (C) LNnT. One-way ANOVA was performed among nitrogen conditions within same strain and same nitrogen conditions. Tukey’s post-hoc test was performed for multiple comparisons between two carbohydrate conditions. Adjusted P < 0.05 * was regarded significant difference.

#
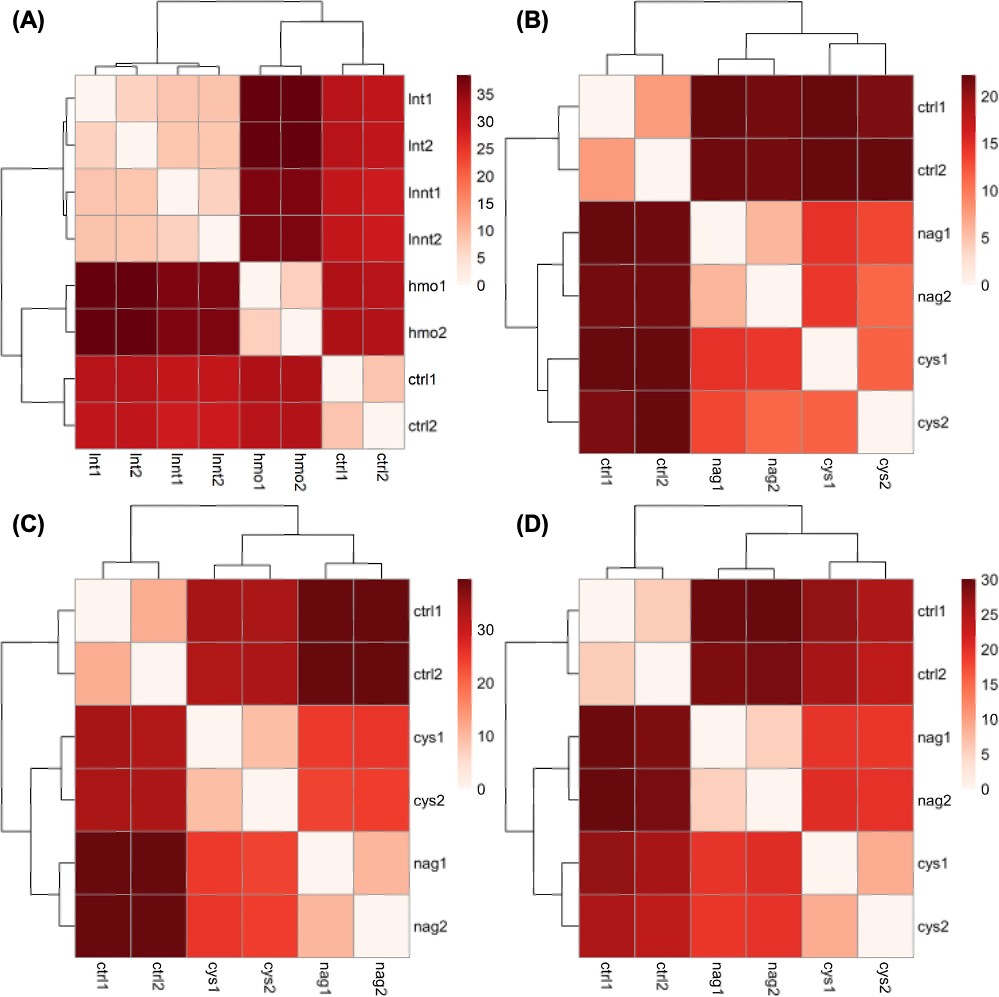
Figure S8

Sample-to-sample distance measured by Euclidian distance for (A) *B. infantis* UMA 272 when subsisting on LNT (lnt1, lnt2), LNnT (lnnt1, lnnt2), pooled HMO (hmo1, hmo2), and complex nitrogen (ctrl1, ctrl2); (B) *B. infantis* UMA 272 when subsisting on NAG (nag1, nag2), L- cysteine (cys1, cys2), and complex nitrogen; (C) *B. infantis* UMA 299 when subsisting on NAG, L-cysteine, and complex nitrogen. (D) *B. infantis* UMA 302 when subsisting on NAG, L-cysteine, and complex nitrogen. Biological replicates were depicted as 1 and 2.

#
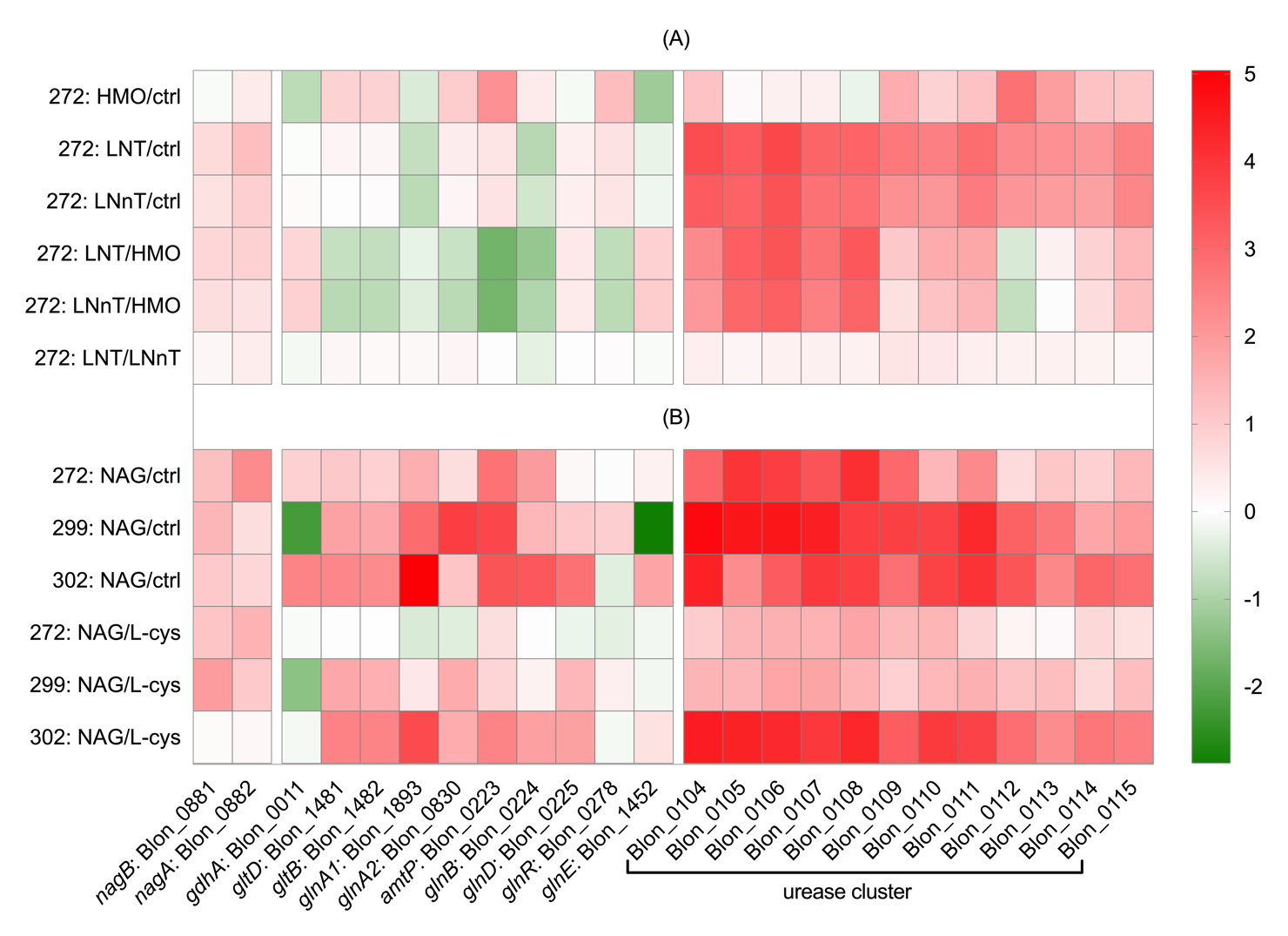
Figure S9

*B. infantis* nitrogen assimilation related gene expressions under pooled HMO, LNT, LNnT, and NAG nitrogen conditions compared to complex nitrogen as control, and NAG compared to L-cysteine. Log2FC values and corresponding false discovery rates (FDR, adjusted p value) are reported in Table S2 and Table S3.

#
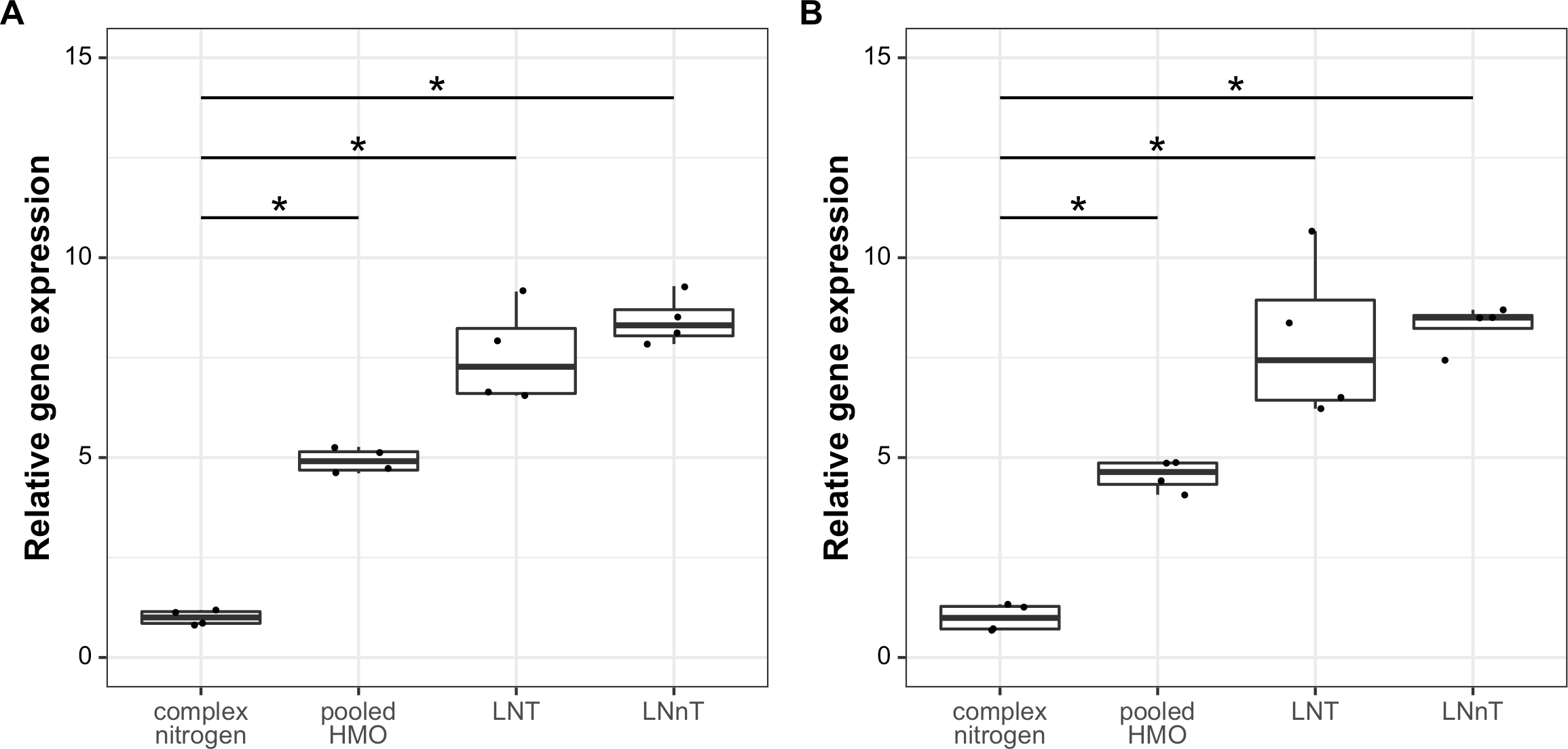
Figure S10

*B. infantis* relative gene expression level in complex nitrogen, pool HMO, LNT, and LNnT nitrogen conditions. (A) Blon_0881 (*nagB*) and (B) Blon_0882 (*nagA*) in *B. infantis* UMA 272, as measured by qRT- PCR. One-way ANOVA was performed among nitrogen conditions within same strain and same carbohydrate conditions. Tukey’s post-hoc test was performed for multiple comparisons between two nitrogen conditions. Adjusted P <0.05 * was regarded significant difference.

#
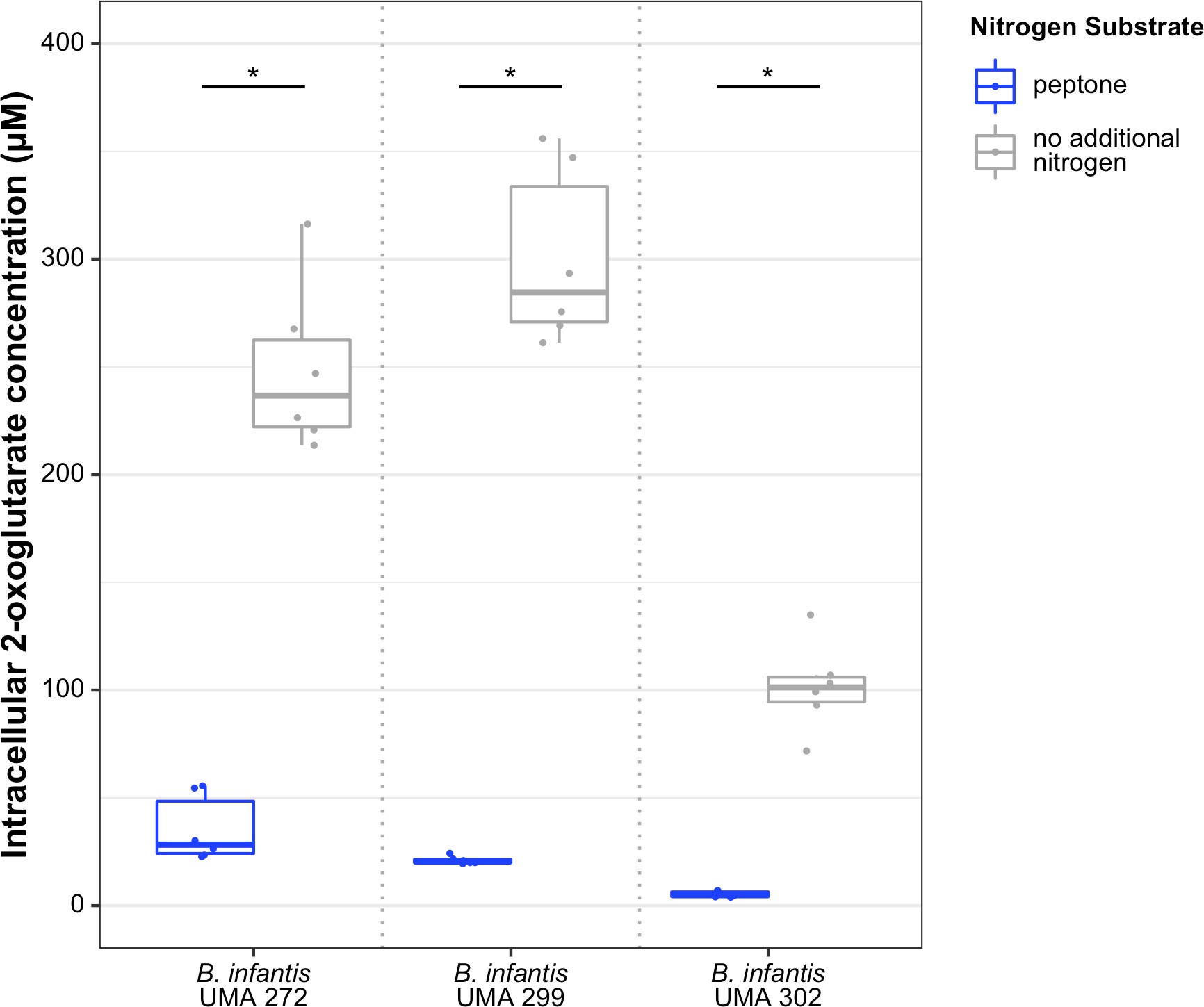
Figure S11

*B. infantis* intracellular 2-oxoglutarate (2- OG) levels peptone and no additional nitrogen (0.03% w/v, L-cysteine) conditions. One-way ANOVA was performed among nitrogen conditions within same strain. Adjusted P <0.05 * was regarded significant difference.
